# Prevalence and trends of developmental disabilities among US children and adolescents aged 3 to 17 years, 2018–2021

**DOI:** 10.1038/s41598-023-44472-1

**Published:** 2023-10-12

**Authors:** Qian Li, Yanmei Li, Juan Zheng, Xiaofang Yan, Jitian Huang, Yingxia Xu, Xia Zeng, Tianran Shen, Xiaohui Xing, Qingsong Chen, Wenhan Yang

**Affiliations:** 1 Department of Child and Adolescent Health, School of Public Health, Guangdong Pharmaceutical University, Guangzhou, 510006 Guangdong Province China; 2 Department of Child Health, Maternity and Child Health Hospital of Baiyun District, Guangzhou, 510400 China; 3 Department of Nutrition and Food Health, School of Public Health, Guangdong Pharmaceutical University, Guangzhou, 510006 Guangdong Province China; 4 Department of Occupational and Environmental Health, School of Public Health, Guangdong Pharmaceutical University, Guangzhou, 510006 Guangdong Province China

**Keywords:** Neuroscience, Psychology

## Abstract

Developmental disabilities prevalence seem to be high in countries around the world. It’s worth understanding the most recent prevalence and trends of developmental disabilities. The objective of this study is to examine the prevalence and trends of developmental disabilities of US children and adolescents. A total of 26,422 individuals aged 3–17 years were included. Annual data were examined from the National Health Interview Survey (2018–2021). Weighted prevalence for each of the selected developmental disabilities were calculated. The prevalence of any developmental disabilities in individuals was 16.65% (95% CI 16.03–17.26%), prevalence of attention deficit/hyperactivity disorder (ADHD), learning disability (LD), autism spectrum disorder (ASD), intellectual disability (ID), and other developmental delay were 9.57% (95% CI 9.09–10.06%), 7.45% (95% CI 7.00–7.89%), 2.94% (95% CI 2.67–3.21%), 1.72% (95% CI 1.51–1.93%), and 5.24% (95% CI 4.89–5.59%), respectively. Significant increases were observed for other developmental delay (4.02–6.05%) and co-occurring LD & ID (1.03–1.82%). Findings form this study highlight a high prevalence of any developmental disabilities, although no significant increase was observed. The prevalence of other developmental delay and co-occurring LD & ID were significantly increased. Further investigation is warranted to assess potentially modifiable risk factors and causes of developmental disabilities.

## Introduction

As a group of lifelong disorders, developmental disabilities are characterized by difficulties in one or more areas, including physical, learning, language, or behavior^1–3^. Examples of more common developmental disabilities include, attention deficit/hyperactivity disorder (ADHD), learning disability (LD), autism spectrum disorder (ASD), intellectual disability (ID), and other developmental delay^4^. Compared to children without developmental disabilities, children with developmental disabilities are at greater risk of suboptimal health, educational attainment and well-being^4^.

Developmental disabilities have profound impacts on the quality of life and social integration of individuals^4^. ADHD is characterized by inattention, hyperactivity, and impulsivity. Individuals with ADHD face an increased risk of adverse outcomes^5–7^. These outcomes include challenges such as decreased educational attainment, higher unemployment rates, increased driving accidents, and elevated risk for other mental health conditions^5–7^. LD primarily refers to difficulties in acquiring academic skills, such as reading or mathematics, which may hinder a child's educational progress^8^. ASD involves challenges in social interaction, communication, and repetitive behaviors, which can lead to differences in forming relationships and navigating everyday activities and social situation^9^. ID is characterized by limitations in intellectual functioning and adaptive behavior, affecting an individual's ability to independently handle daily tasks^10^. Other developmental delay encompasses a range of issues not falling into the above categories but still requiring specialized attention and support^1–3^.

It is important to note that, the number of children with developmental disabilities has been increasing all around the world in the past decades^4^. The United States is one of the major countries in the world with significant increasing trends in the prevalence of most developmental disabilities^4^. This can be attributed to various factors, such as improved awareness and identification of developmental disabilities, increased access to healthcare services, and changes in diagnostic criteria^1,4^. Over the past decade, the prevalence has steadily increased, with 17.76% in 2015–2017 compared with 16.22% in 2009–2011^1^. From 1997 to 2006, the average annual increase in the percentage of children diagnosed with ADHD was 3%^11^. Another study also showed that the prevalence of diagnosed ADHD among children and adolescents in the US increased from 6.1% in 1997–1998 to 10.2% in 2015–2016^5^. There was no significant annual average change was found in the percentage of children diagnosed with LD from 1997 to 2006^11^. In 2007, 5.5% of all students in public schools were identified as having LD^8^. The results of a recent study suggest that the prevalence of ASD among individuals aged 3 to 17 years in the US, based on data from the 2019 and 2020 survey years of NHIS, is approximately 3.14%^12^. In addition, an analysis of the National Health Interview Survey (NHIS) from 2014 to 2016 reported 23.21%, 3.64%, and 27.45% increase in the prevalence of ASD, ID, and other developmental delay, respectively^13^.

Understanding the prevalence and trends of these developmental disabilities is crucial. Firstly, the adverse impacts of these conditions on individuals and their families are profound and enduring^1,5–10^. They can lead to lower educational attainment, decreased quality of life, and increased healthcare needs^1,5–10^. Secondly, tracking the prevalence of these disabilities over time provides critical insights into potential factors such as changes in diagnostic criteria, awareness, or environmental influences^1,2^.

According to previous studies, it was common for children to be diagnosed with two or more of developmental disabilities^9,11,14^. This situation can complicate diagnosis and treatment, further impacting their quality of life and leading to economic burdens^9,11,14^. ADHD, LD, ASD, and ID are common co-occurring developmental disabilities^9,11,14^. For instance, approximately 30% to 80% of children with ASD also meet criteria for ID, and the estimated prevalence of co-occurring ASD and ADHD in children ranges from 20 to 50%^14^. The massive range may be due to studies conducted in various regions, cultures, and communities that may include individuals with varying degrees of developmental disabilities^9,11,14^. However, at present, studies on co-occurring developmental disabilities are still lacking. Current research predominantly focuses on a single condition, however, for individuals concurrently affected by multiple conditions, better comprehension of the risk factors leading to the co-occurrence of these conditions is needed. Understanding these risk factors can assist in better prevention and intervention strategies for co-occurring developmental disabilities.

Information on the prevalence and trends of developmental disabilities in recent years can provide important information for future research, clinical care, and decision-making about developmental disabilities. However, epidemiological data on developmental disabilities to guide comprehensive health policy engagements at the regional or national levels are lacking^4^. Timely data are not only critical to capturing changes in the prevalence of developmental disabilities, but also provide an opportunity to address challenges in estimating prevalence. The primary objective of this report is to describe the prevalence and the trends of developmental disabilities in individuals aged 3–17 years in the US using timely, nationally representative data from NHIS. As a secondary objective, this study also examined the prevalence of all possible combinations of two or more co-occurring ADHD, LD, ASD, and ID. These combinations include co-occurring ADHD & LD, co-occurring LD & ASD, co-occurring LD & ID, co-occurring ADHD & ASD, co-occurring ASD & ID, co-occurring ADHD & ID, co-occurring ADHD & LD & ASD, co-occurring LD & ASD & ID, co-occurring ADHD & LD & ID, co-occurring ADHD & ASD & ID, and co-occurring ADHD & LD & ASD & ID.

## Methods

### Data source

Data from the 2018–2021 NHIS, a nationally representative cross-sectional survey of the civilian non-institutionalized U.S. population, were used for this analysis. As a leading national health survey in the US, NHIS is conducted annually by the National Center for Health Statistics at the Centers for Disease Control and Prevention^5^. The NHIS is conducted using computer-assisted personal interviewing^5^. Face-to-face interviews are conducted in respondents’ homes, but follow-ups to complete interviews may be conducted over the telephone^6^. According to the data collection procedures of NHIS (https://www.cdc.gov/nchs/nhis/about_nhis.htm), a telephone interview may be conducted when the respondent requests a telephone interview or when road conditions or travel distances would make it difficult to schedule a personal visit before the required completion date. Information about the sample child was collected by interviewing a parent or guardian, who was knowledgeable about the child’s health^5^. For each interviewed family in the household, only one sample child, if any, was randomly selected by a computer program^5^. Sample children aged 3–17 years were included in this analysis (total unweighted sample size: n = 26,422). The total household response rate ranged from 50.7% to 64.2% and the conditional response rate for the sample child ranged from 86.9% to 93.5%, between 2018 and 2021.

### Measures

Selected demographic and socioeconomic characteristics were provided by NHIS, including child’s age, sex, race/ethnicity, highest educational level of family members, family income to poverty ratio, and geographic region, collected during the interview using a standardized questionnaire. Race and Hispanic ethnicity (Hispanic, non-Hispanic White, non-Hispanic Black, and Other) were self-reported and classified based on the 1997 Office of Management and Budget Standards^5^. Income levels (family income to poverty ratio < 1.00, 1.00 to 1.99, 2.00 to 3.99, and ≥ 4.00) were classified according to the ratio of family income to federal poverty level^5^. In order to better understand the characteristics, needs, and developmental trends of children and adolescents of different age groups, we divided age into 3–5 years old (preschools), 6–11 years old (children), and 12–17 years old (adolescents). Following previous research, we categorized the highest educational level of family members into Less than high school, High school, and College or higher, in order to have a clear and concise understanding and analysis of the educational background of family members^12^. The classification of geographic region was determined by NHIS and divided into Northeast, Midwest, South, and West.

Developmental disabilities examined in this report were ADHD, LD, ASD, ID, and other developmental delay. ADHD, LD, ASD, ID, and other developmental delay were defined according to affirmative responses to the questions listed in Table S of Supplement respectively. In this study, parents were asked whether a doctor or health professional had ever informed them that their child had any of these conditions (ASD, ADHD, LD, ID, and other developmental delay), and children whose parents responded affirmatively to any of these conditions were defined as cases. A child was categorized as having “any developmental disability” if they presented with one or more of these aforementioned conditions at any point during their lifetime.

### Statistical analysis

We estimated the prevalence estimates using survey weights, strata, and primary sampling units created by the National Center for Health Statistics to account for unequal probabilities of selection, oversampling, and nonresponse in the survey.

Differences between percentages of developmental disabilities by selected demographic and socioeconomic characteristics were evaluated using chi-squares. Given that prevalence may vary over time, we tested trends in the prevalence over time using a weighted logistic regression model, which included the survey year as a continuous variable and adjusted for age, sex, race/ethnicity, highest educational level of family members, family income, and geographic region. Prevalence and trends of co-occurring two or more of ADHD, LD, ASD, and ID were also examined. All statistical analyses were performed using SAS, version 9.4 (SAS Institute, Inc.). Two-sided *P* < 0.05 was considered statistically significant.

All methods were carried out in accordance with the Declaration of Helsinki. NHIS was approved by the National Center for Health Statistics Research Ethics Review Board. All respondents provided informed verbal consent prior to participation. The Guangdong Pharmaceutical University Academic Review Board determined the present study was exempt from approval because of the use of de-identified data.

### Ethical approval

The NHIS protocols were approved by the National Center for Health Statistics research ethics review board. Written informed consent was obtained for all participants. The Guangdong Pharmaceutical University Academic Review Board determined the present study was exempt from approval because of the use of deidentified data.

## Results

Among the total of 26,422 individuals aged 3–17 years included in the study, comprising 13,612 boys (51.52%) and 12,810 girls (48.48%), the overall prevalence of any developmental disability reported by parents was 16.65% (95% CI 16.03–17.26%) from 2018 to 2021. Specifically, the prevalence was 16.19% (95% CI 15.04–17.35%) in 2018, 15.42% (95% CI 14.45–16.40%) in 2019, 17.91% (95% CI 16.54–19.28%) in 2020, and 17.07% (95% CI 16.05–18.09%) in 2021. There was no significant trend observed over time (*P* for trend = 0.22). From 2018 to 2021, significant variations in the prevalence of any developmental disability were noted across different age groups, sexes, races/ethnicities, family income-to-poverty ratios, and geographic regions (Table 1).

**Table 1:** Trends in the Prevalence of any developmental disability in US Children and Adolescents aged 3 to 17 years, 2018–2021.

Characteristic	Prevalence, % (95% CI)^b^				*P* for trend	Prevalence, % (95% CI)^b^	*P* value^c^
	2018	2019	2020	2021		2018–2021	
No. of participants overall^a^	6939	7684	4870	6929	–	26,422	–
No. of participants with any developmental disability^a^	1166	1260	877	1225		4528	
Overall prevalence prevalence	16.19 (15.04–17.35)	15.42 (14.45–16.40)	17.91 (16.54–19.28)	17.07 (16.05–18.09)	0.22	16.65 (16.03–17.26)	–
Age, year							
3–5	8.01 (6.07–9.96)	9.61 (7.81–11.41)	10.15 (7.59–12.71)	9.41 (7.35–11.48)	0.45	9.29 (8.28–10.30)	< 0.001
6–11	16.08 (14.37–17.80)	15.72 (13.99–17.46)	17.80 (15.42–20.19)	16.60 (14.87–18.32)	0.70	16.54 (15.56–17.52)	
12–17	20.33 (18.57–22.10)	17.91 (16.40–19.41)	21.59 (19.35–23.83)	21.12 (19.51–22.74)	0.24	20.24 (19.26–21.22)	
Sex							
Male	20.86 (19.08–22.64)	20.12 (18.69–21.56)	22.20 (20.17–24.23)	21.65 (20.05–23.26)	0.87	21.21 (20.30–22.12)	< 0.001
Female	11.39 (10.07–12.71)	10.54 (9.33–11.76)	13.38 (11.65–15.12)	12.32 (11.12–13.52)	0.06	11.90 (11.21–12.59)	
Race/ethnicity^d^							
Hispanic	14.56 (12.41–16.72)	12.74 (11.04–14.44)	15.17 (12.52–17.81)	14.31 (12.43–16.19)	0.95	14.19 (13.13–15.26)	< 0.001
Non-Hispanic white	16.95 (15.47–18.43)	17.36 (15.99–18.73)	19.97 (18.07–21.87)	18.94 (17.44–20.44)	0.04	18.31 (17.46–19.16)	
Non-Hispanic black	18.25 (14.61–21.89)	15.79 (12.78–18.01)	19.21 (14.58–23.85)	18.44 (14.97–21.90)	0.94	17.91 (16.02–19.80)	
Other^e^	13.91 (10.78–17.05)	11.89 (9.32–14.46)	12.09 (8.84–15.34)	13.34 (10.73–15.94)	0.43	12.86 (11.37–14.35)	
Highest educational level of family members							
Less than high school	15.82 (12.25–19.39)	15.41 (11.55–19.27)	17.38 (11.97–22.78)	17.13 (12.75–21.50)	0.70	16.33 (13.97–18.69)	0.51
High school	16.43 (13.73–19.13)	17.02 (14.53–19.51)	18.43 (14.61–22.24)	17.79 (15.13–20.45)	0.51	17.42 (15.82–19.01)	
College or higher	16.24 (14.92–17.57)	15.07 (14.00–16.15)	17.87 (16.38–19.37)	16.91 (15.77–18.06)	0.32	16.54 (15.87–17.21)	
Missing	–	19.01 (0.00–51.37)	–	11.78 (0.00–33.69)	< 0.001	7.05 (0.00–17.28)	
Family income to poverty ratio^g^							
< 1.00	21.79 (18.20–25.37)	20.08 (17.23–22.92)	24.58 (20.10–29.06)	21.12 (18.04–24.21)	0.78	21.82 (20.03–23.60)	< 0.001
1.00–1.99	18.62 (15.84–21.39)	16.08 (14.01–18.14)	18.26 (15.28–21.24)	18.85 (16.54–21.15)	0.86	17.92 (16.60–19.24)	
2.00–3.99	15.82 (13.56–15.54)	14.43 (12.77–16.10)	15.25 (12.97–17.54)	15.96 (14.20–17.71)	0.69	15.34 (14.36–16.31)	
≥ 4.00	13.80 (12.06–15.54)	13.21 (11.65–14.77)	16.95 (14.76–19.13)	14.74 (13.09–16.38)	0.12	14.70 (13.73–15.67)	
Missing	13.84 (11.28–16.40)	–	–	–	–	13.84 (11.29–16.40)	
Geographic region							
Northeast	17.10 (13.24–20.96)	15.18 (12.44–17.92)	19.80 (16.62–22.98)	17.12 (14.41–19.83)	0.62	17.28 (15.61–18.95)	< 0.001
Midwest	16.97 (14.71–19.24)	16.00 (13.60–18.39)	19.60 (16.50–22.70)	16.15 (13.99–18.32)	0.96	17.19 (15.82–18.55)	
South	17.76 (15.90–19.63)	16.89 (15.38–18.39)	18.33 (15.98–20.68)	18.82 (17.07–20.57)	0.57	17.95 (16.92–18.98)	
West	12.66 (10.77–14.54)	12.79 (11.17–14.42)	14.67 (12.08–17.25)	14.97 (13.24–16.70)	0.14	13.75 (12.70–14.81)	

^a^The number of participants overall and with any developmental disability were unweighted.

^b^Prevalence estimates were weighted.

^c^*P* values were estimated for the difference in prevalence by strata.

^d^Race and ethnicity were self-reported and classified based on the 1997 Office of Management and Budget standards.

^e^Other races and ethnicities included non-Hispanic American Indian or Alaska Native individual only, non-Hispanic American Indian or Alaska Native and any other group, non-Hispanic Asian individual only, and other single and multiple races, or declined to respond, no response, or unknown.

^g^The ratio is the total family income divided by the poverty threshold.

The weighted prevalence of ADHD, LD, ASD, ID, and other developmental delay were 9.57% (95% CI 9.09-10.06%), 7.45% (95% 7.00-7.89%), 2.94% (95% CI 2.67–3.21%), 1.72% (95% CI 1.51–1.93%), and 5.24% (95% CI 4.89–5.59%), respectively (Table 2).

**Table 2:** Prevalence of developmental disabilities in US children and Adolescents aged 3–17 years, 2018–2021.

Characteristic	Total^a^	ADHD			LD			ASD		
		n^a^	% (95% CI)^b^	*P* value^c^	n^a^	% (95% CI)^b^	*P* value^c^	n^a^	% (95% CI)^b^	*P* value^c^
Overall prevalence	26,422	2679	9.57 (9.09–10.06)	–	1986	7.45 (7.00–7.89)	–	811	2.94 (2.67–3.21)	–
Age, year										
3–5	4652	68	1.53 (1.11–1.94)	< 0.001	153	3.40 (2.74–4.06)	< 0.001	110	2.09 (1.63–2.55)	0.005
6–11	9588	956	9.59 (8.80–10.37)		713	7.50 (6.81–8.20)		322	3.25 (2.77–3.74)	
12–17	12,182	1655	13.39 (12.58–14.19)		1120	9.31 (8.58–10.03)		379	3.04 (2.63–3.45)	
Sex										
Male	13,612	1804	12.41 (11.69–13.13)	< 0.001	1256	9.25 (8.57–9.94)	< 0.001	626	4.37 (3.93–4.82)	< 0.001
Female	12,810	875	6.63 (6.09–7.16)		730	5.57 (5.06–6.08)		185	1.45 (1.19–1.71)	
Race/ethnicity^d^										
Hispanic	6341	469	7.19 (6.44–7.94)	< 0.001	486	7.50 (6.72–8.27)	< 0.001	182	2.79 (2.31–3.27)	0.24
Non-Hispanic white	13,940	1679	11.12 (10.46–11.79)		1079	7.40 (6.81–7.99)		442	2.86 (2.54–3.17)	
Non-Hispanic black	2891	315	10.50 (9.01–11.99)		251	9.20 (7.65–10.74)		98	3.69 (2.54–4.84)	
Other^e^	3250	216	6.61 (5.56–7.66)		170	5.31 (4.40–6.22)		89	2.79 (2.06–3.52)	
Highest educational level of family members										
Less than high school	1821	175	8.68 (6.98–10.39)	0.46	198	9.95 (8.12–11.77)	< 0.001	48	2.19 (1.36–3.03)	0.11
High school	3930	428	10.22 (9.04–11.41)		361	8.93 (7.75–10.11)		114	2.64 (2.04–3.24)	
College or higher	20,643	2074	9.54 (9.00–10.09)		1426	6.88 (6.40–7.35)		649	3.09 (2.77–3.40)	
Missing	28	2	7.05 (0.00–17.28)		1	2.29 (0.00–6.83)		0	–	
Family income to poverty ratio^g^										
< 1.00	3161	442	12.71 (11.25–14.18)	< 0.001	404	11.93 (10.51–13.35)	< 0.001	139	4.32 (3.39–5.25)	< 0.001
1.00–1.99	5184	567	10.26 (9.26–11.26)		467	8.46 (7.45–9.47)		185	2.99 (2.43–3.55)	
2.00–3.99	7793	730	8.39 (7.64–9.13)		529	6.41 (5.69–7.13)		252	3.11 (2.56–3.67)	
≥ 4.00	9290	851	8.75 (8.03–9.47)		517	5.57 (4.96–6.18)		215	2.21 (1.84–2.58)	
Missing	994	89	8.42 (6.34–10.50)		69	6.26 (4.44–8.08)		20	1.56 (0.82–2.31)	
Geographic region										
Northeast	4175	394	9.11 (7.91–10.32)	< 0.001	362	8.65 (7.49–9.81)	0.01	155	3.95 (3.05–4.85)	0.02
Midwest	5590	593	10.02 (8.83–11.21)		412	7.16 (6.25–8.07)		163	2.92 (2.28–3.56)	
South	9628	1191	11.26 (10.43–12.09)		748	7.74 (6.97–8.52)		299	2.66 (2.30–3.01)	
West	7029	501	6.87 (6.12–7.62)		464	6.45 (5.63–7.26)		194	2.75 (2.24–3.27)	

Characteristic	Total^a^	ID			Other developmental delay					
		n^a^	% (95% CI)^b^	*P *value^c^	n^a^	% (95% CI)^b^	*P *value^c^			
Overall prevalence	26422	446	1.72 (1.51–1.93)	–	1331	5.24 (4.89–5.59)	–			
Age, year										
3–5	4652	49	0.95 (0.65–1.26)	< 0.001	279	6.31 (5.42–7.19)	0.002			
6–11	9588	151	1.77 (1.39–2.15)		514	5.34 (4.77–5.91)				
12–17	12182	246	2.03 (1.71–2.35)		538	4.63 (4.16–5.11)				
Sex										
Male	13612	282	2.16 (1.82–2.49)	< 0.001	867	6.76 (6.21–7.32)	< 0.001			
Female	12810	164	1.26 (1.03–1.49)		464	3.65 (3.25–4.06)				
Race/ethnicity^d^										
Hispanic	6341	110	1.70 (1.31–2.08)	< 0.001	296	4.74 (4.02–5.47)	0.04			
Non-Hispanic white	13940	221	1.57 (1.31–1.84)		745	5.49 (5.00–5.97)				
Non-Hispanic black	2891	73	2.75 (1.87–3.63)		147	6.02 (4.79–7.25)				
Other^e^	3250	42	1.20 (0.80–1.59)		143	4.25 (3.42–5.07)				
Highest educational level of family members										
Less than high school	1821	45	2.22 (1.45–2.98)	0.19	95	5.00 (3.73–6.27)	0.59			
High school	3930	76	1.90 (1.39–2.41)		188	4.86 (3.97–5.74)				
College or higher	20643	325	1.63 (1.40–1.86)		1048	5.35 (4.95–5.75)				
Missing	28	0	–	–	0	–	–			
Family income to poverty ratio^g^										
< 1.00	3161	88	2.51 (1.88–3.15)	< 0.001	198	6.25 (5.18–7.32)	0.001			
1.00–1.99	5184	115	2.17 (1.65–2.69)		297	5.98 (5.16–6.80)				
2.00–3.99	7793	110	1.47 (1.09–1.85)		383	4.97 (4.37–5.57)				
≥ 4.00	9290	119	1.28 (1.00–1.57)		408	4.69 (4.11–5.27)				
Missing	994	14	1.26 (0.52–2.00)		45	3.29 (2.08–4.50)				
Geographic region										
Northeast	4175	84	2.35 (1.71–3.00)	0.08	238	6.21 (5.22–7.21)	0.10			
Midwest	5590	90	1.51 (1.10–1.92)		286	5.16 (4.38–5.95)				
South	9628	166	1.62 (1.32–1.91)		475	5.16 (4.60–5.71)				
West	7029	106	1.65 (1.18–2.11)		332	4.81 (4.16–5.45)				

*ADHD* attention-deficit/hyperactivity disorder, *LD* learning disability, *ASD* autism spectrum disorder, *ID* intellectual disability.

^a^Unweighted number of participants.

^b^Prevalence estimates were weighted.

^c^*P* values were estimated for the difference in prevalence by strata.

^d^Race and ethnicity were self-reported and classified based on the 1997 Office of Management and Budget standards.

^e^Other races and ethnicities included non-Hispanic American Indian or Alaska Native individual only, non-Hispanic American Indian or Alaska Native and any other group, non-Hispanic Asian individual only, and other single and multiple races, or declined to respond, no response, or unknown.

^g^The ratio is the total family income divided by the poverty threshold.

**Figure 1:**
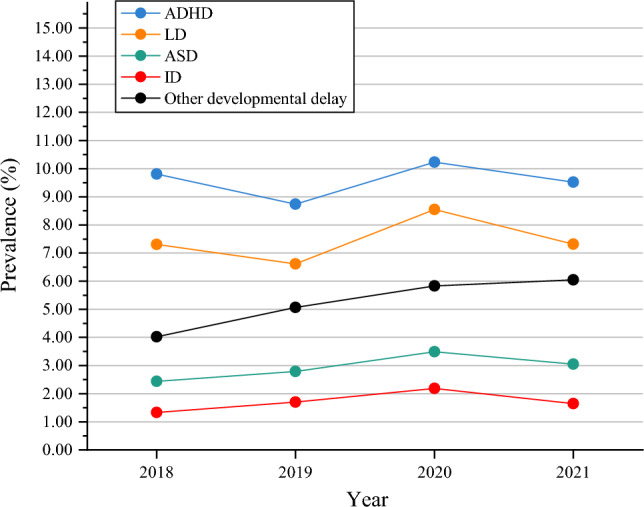
Trends in the Prevalence of Developmental Disabilities in US Children and Adolescents aged 3–17 years, 2018–2021. *ADHD* attention-deficit/hyperactivity disorder; *LD* learning disability; *ASD* autism spectrum disorder; *ID* intellectual disability.

**Figure 2:**
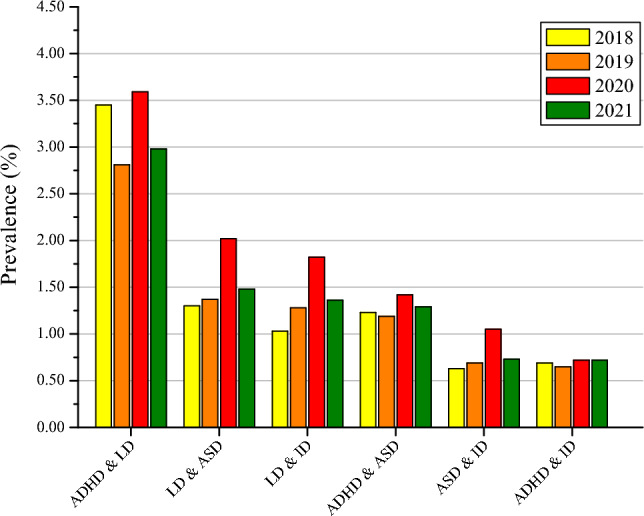
Prevalence of children and adolescents aged 3–17 years with two of selected developmental disabilities, by year: US, 2018–2021. ADHD & LD: co-occurring ADHD & LD; LD & ASD: co-occurring LD & ASD; LD & ID: co-occurring LD & ID; ADHD & ASD: co-occurring ADHD & ASD; ASD & ID: co-occurring ASD & ID; ADHD & ID: co-occurring ADHD & ID.

**Figure 3:**
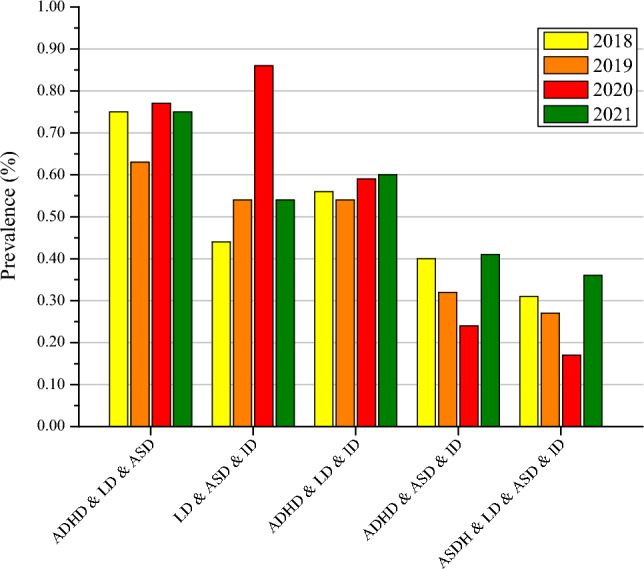
Prevalence of children and adolescents aged 3–17 years with three or more of selected developmental disabilities, by year: US, 2018–2021. The vertical label refers from left to right: co-occurring ADHD & LD & ASD, co-occurring LD & ASD & ID, co-occurring ADHD & LD & ID, co-occurring ADHD & ASD & ID, and co-occurring ADHD & LD & ASD & ID.

Other developmental delay exhibited the only statistically significant increase in prevalence from 2018 to 2021 (4.02%, 95% CI 3.46–4.58%; 6.05%, 95% CI 5.36–6.74%; *p* < 0.05, respectively) (Fig. 1). Moreover, an analysis of the co-occurring developmental disabilities among individuals aged 3 to 17 in the United States from 2018 to 2021 revealed significant overall increases in the prevalence of co-occurring LD & ID (1.03–1.82%, *P* for trend < 0.05) (Fig. 2). There were not significant increases observed for ADHD, LD, ASD, ID, co-occurring ADHD & LD, co-occurring LD & ASD, co-occurring ADHD & ASD, co-occurring ASD & ID, co-occurring ADHD & ID, co-occurring ADHD & LD & ASD, co-occurring LD & ASD & ID, co-occurring ADHD & LD & ID, co-occurring ADHD & ASD & ID, and co-occurring ADHD & LD & ASD & ID, all *P* for trend > 0.05 (Figs. 1, 2 and 3).

Data from the 2018–2021 survey years of NHIS indicated that the prevalence of ADHD, LD, ASD, or ID were generally higher among the 12–17 years age group, boys, non-Hispanic white individuals or non-Hispanic black individuals, and individuals with lower family income levels. Additionally, the prevalence of other developmental delay was higher among the 3–5 years age group, boys, non-Hispanic black individuals, and individuals with lower family income levels. (see Supplementary Fig. S1–S5 online).

From 2018 to 2021, the prevalence of ADHD ranged from 11.95% to 13.64% in individuals aged 12 to 17 years, 11.68–12.92% in boys, from 10.26 to 11.90% in non-Hispanic white individuals, and from 11.19 to 14.92% in family income-to-poverty ratio less than 1.00 (see Supplementary Fig. S1 online). The prevalence of LD ranged from 8.11 to 10.61% in individuals aged 12 to 17 years, 11.68–12.92% in boys, from 8.59 to 9.80% in non-Hispanic white individuals, and from 11.19 to 14.92% in family income-to-poverty ratio less than 1.00 (see Supplementary Fig. S2 online). The prevalence of ASD ranged from 2.41 to 3.88% in individuals aged 12 to 17 years, 3.54 to 4.98% in boys, from 2.08 to 5.22% in non-Hispanic black individuals, and from 3.21 to 6.21% in family income-to-poverty ratio less than 1.00 (see Supplementary Fig. S3 online). As for ID, the prevalence ranged from 1.49 to 2.51% in children aged 12 to 17 years, from 1.70 to 2.76% in boys, from 1.95 to 4.29% in non-Hispanic black individuals, and from 2.20 to 2.38% in family income-to-poverty ratio less than 1.00 (see Supplementary Fig. S4 online). From 2018 to 2021, significant increases in prevalence of other developmental delay were observed for children aged 6 to 11 years (3.94–6.38%), adolescents aged 12 to 17 years (3.87–5.36%), boys (5.18–7.63%), and non-Hispanic black individuals (4.05–7.70%), in addition, the prevalence of other developmental delay ranged from 5.11 to 7.45% among individuals with a family income-to-poverty ratio less than 1.00 (see Supplementary Fig. S5 online).

## Discussion

This study provided the parent-reported prevalence estimates of ADHD, LD, ID, ASD, and other developmental delay in children and adolescents aged 3–17 years. The overall prevalence of any developmental disability did not increase significantly during this period. Approximately one in six children and adolescents aged 3–17 years in the US were reported by their parents to have any developmental disability during the period of 2018–2021. Additionally, the latest data from NHIS indicated that prevalence estimates of ADHD, LD, ASD, and ID were mostly similar to those reported in previous years. Moreover, we also found that the prevalence of other developmental delay and co-occurring LD & ID were significantly increased.

From the results, we observed that the prevalence of developmental disabilities in 2021 was lower than that in 2020. The COVID-19 pandemic may have indirectly influenced the prevalence of developmental disabilities by affecting people's lifestyles, healthcare access, and social support systems^15^. For instance, since the onset of the pandemic, disruptions in services for children with developmental disabilities in the United States and a transition to remote healthcare have been widespread^15^. While most developmental disabilities (ADHD, LD, ASD, and ID) did not show an increasing trend during the period of this study from 2018 to 2021, the prevalence of developmental disabilities appeared to be higher compared to findings from previous research. It should be noted that changes in diagnostic criteria may have an impact on the reported overall prevalence rates provided in this study. DSM-5, the fifth edition of the Diagnostic and Statistical Manual of Mental Disorders, was implemented in 2013, replacing DSM-IV^16,17^. For developmental disabilities, DSM-5 introduced significant changes to the diagnostic criteria, which could affect the prevalence rates^16,17^. For instance, DSM-5 revised the age range and subtype diagnosis for ADHD, merged the diagnoses of ASD into a unified concept, and made adjustments to the classification and assessment of LD and ID^16,17^. These changes may have an impact on the prevalence rates, and further research and data are needed to evaluate the specific effects.

### Attention-deficit/hyperactivity disorder

The overall weighted prevalence of ADHD in 2018–2021 was 9.57% and there was not a significant increase in the prevalence of ADHD during this period. The prevalence in this study was higher than the community prevalence globally (ranged from 2 to 7%) from a systematic review in 2018^18^, very similar to the results from 2015–2017 (9.54%) to 2017–2018 (9.6%) of NHIS^1,6^, and slightly lower than the prevalence reported from 2016 to 2019 (9.8%) of National Survey of Children’s Health (NSCH)^6^. In addition, this study found that the prevalence of co-occurring ADHD & LD was similar to the 3.7% prevalence of both conditions in children and adolescents aged 6 to 17 reported by the NHIS from 2004 to 2006^11^. The annual prevalence of ADHD has not changed much in recent years. Notably, the estimated prevalence of ADHD among U.S. individuals in this study was higher than the prevalence estimated in 1997–1998 (6.1%)^19^. In theory, the increased prevalence may be due to increased awareness of ADHD and its different manifestations^19^.

Given that ADHD has a high prevalence that persists into adult life, individuals with this condition face an increased risk of adverse outcomes^6,7^. Thus timely identification and treatment of children and adolescents with ADHD provides an opportunity to improve long-term outcomes. However, as a neurodevelopmental disorder, ADHD has a highly complex etiology. Both genetic risk factors and environmental risk factors are believed to contribute to the development of ADHD^5^. The contributions of these risk factors to the etiologic source of ADHD, both separately and jointly, warrant further investigation.

Moreover, we found that the prevalence of co-occurring ADHD & LD was higher than co-occurring ADHD & other developmental disabilities. It’s not uncommon for individuals with ADHD to also experience co-occurring LD, ASD, or ID^11,14,20^. It is worth noting that the co-occurring ADHD & LD can introduce additional complexity to the educational setting^11^. The presence of ADHD, ASD, and ID can complicate diagnostic and intervention efforts because some symptoms overlap, for example, difficulties with attention and impulsivity are all common symptoms of ADHD, ASD, and ID^14^. This further emphasizes the complexity of developmental disabilities. Further investigation is warranted to assess potentially modifiable risk factors, and provide adequate resources for treatment of affected individuals in the future.

### Learning disability

This study did not find a significant increase in the prevalence of LD among children and adolescents in the US, nor did previous study observe a significant increase in LD prevalence^1^. The observed prevalence of LD was similar to the prevalence in 2015–2018 (7.7%) and in 2015–2017 (7.86%) from NHIS^1,2^. These data indicate that LD is a common chronic condition among US children, affecting about 8 in 100 overall. LD is difficult to diagnose and older children may have higher prevalence rate due to longer exposure to the possibility of evaluation and diagnosis^11^. Notably, the Response to Intervention (RTI) framework is used in US schools to provide early interventions for students' academic and behavioral success^21,22^. By intervening early and offering various levels of support, the RTI framework can potentially result in improved academic outcomes for many students^21,22^. Thus, the RTI framework could have implications for the diagnosis of LD.

This study found that the prevalence of co-occurring LD & ID showed a significant increase trend. Although LD and ID are clinically regarded as distinct conditions, both LD and ID can involve difficulties with learning and academic achievement^8,9,11,14^. This could imply deeper associations, such as shared genetic factors, overlapping neurodevelopmental pathways, and more. Future research will need to delve deeper into this issue, including exploring potential influencing factors, examining relationships between variables, and assessing how this trend might impact clinical practices and intervention strategies. Moreover, LD may prevent children from reaching their full potential and cause educational, social, and economic burdens^8^. The consensus among experts and scholars is that children who exhibit signs of LD should be identified and intervened as early as possible, because the beneficial effects of early identification and intervention are clear^8^.

### Autism spectrum disorder

The parent-reported lifetime prevalence from this study was higher than the reported results in 2014–2016 (2.47%) and in 2017–2018 (2.4%)^6,23^. The observed prevalence was also higher than the prevalence in 2018 (2.30%) from the Autism and Developmental Disabilities Monitoring Network (ADDM), which assesses the prevalence of currently diagnosed children with autism^24^. Additionally, the observed prevalence was higher than the prevalence in 2016 (2.50%) from NSCH^25^, slightly lower than the prevalence in 2016–2019 (3.1%) from NSCH and the prevalence in 2019–2020 (3.14%) from NHIS^6,13^. A review in 2012 commissioned by WHO estimated the global prevalence of ASD at about 1%^26^, and systematic review analysis of the global prevalence of ASD from 2008 to 2022 showed that the prevalence of ASD in Asia, America, Europe, Africa and Australia was 0.4%, 1%, 0.5%, 1%, and 1.7% respectively^27^. Additionally, this study also found that among the co-occurring developmental disabilities investigated in this study, the prevalence of co-occurring LD & ASD, co-occurring ADHD & LD & ASD, and co-occurring LD & ASD & ID were high; and it has been pointed out that the rate of co-occurring ASD and ID is high^14^.

Estimates of prevalence vary from study to study in different countries^27^. Variations in prevalence stem from differences in diagnostic standards and practices, cultural influences, healthcare access, study designs and protocols, and awareness levels^27^. ASD primarily impacts social communication and behavior habits, and may affect the typical development of children and adolescents^27,28^. Considering ASD is a lifetime diagnosis, parents of children with ASD face addition challenges in ensuring their child is provided with treatment, services, and necessary resources^26–28^. There are also varying degrees of ASD and some children with ASD may not be able to live independently which places additional strain on caregivers, healthcare systems, and the economy^26–28^. Study suggested that total costs attributable to ASD will rise to more than 450 billion dollars by 2025^28^. It is common for ASD to co-occur with other developmental disabilities. ASD may be caused by the interaction of genetic and environmental factors, that is, genetic abnormalities cause a particular individual's genetic susceptibility, and adverse environmental factors before conception, during pregnancy, or during childbirth are the trigger factors for the condition^27,29^. The contribution of these non-genetic and genetic risk factors to the etiological source of ASD deserves further investigation.

### Intellectual disability

The results of this study indicated no significant increase in the prevalence of individuals diagnosed with ID from 2018 to 2021, but the overall prevalence was higher than in previous studies. In this study, the overall prevalence of ID was higher than that of the (ADDM) among eight-year-old children in 2014 (1.2%) and the reported prevalence in 2015–2017 (1.17%) from NHIS^1,10^. In 2019, systematic review reported the prevalence ranged from 1.10 to 1.34% in children and adolescents with ID in 2010 or later, a range smaller than the prevalence reported by this study (1.33–2.19%)^30^. It is worth noting that past increases in the prevalence of ID appear to be related to changes in the terminology of survey questions in the NHIS. From 1997 to 2010, when the survey asked about “mental retardation”, the NHIS has updated the term to “intellectual disability”, also known as “mental retardation” since 2011^30^. The prevalence of ID was relatively stable (7.1 or 7.8 per 1000) during the 1997–2010 study period, but increased by 72% in 2011–2013^1,30^. Although this study used the same terminology, the prevalence of ID described in this report was higher than that described in previous reports using NHIS data^1,2,30^. As a severe lifelong disability, the most common causes of ID are birth defects and genetic conditions. In addition, the risk of ID is increased by older maternal age at childbirth, lower maternal education, lower socioeconomic status and so on^10^. Future studies could further explore the interactions among different factors to gain a more comprehensive insight into the mechanisms and preventative measures associated with intellectual disabilities.

### Other developmental delay

In this study, other developmental delay showed a statistically significant increase over time, contrary to a statistically significant decrease in 2009 to 2017 reported by Zablotsky, et al.^1^. The possible reason for the reduced prevalence seen in the study by Zablotsky and colleagues was that children have increasingly been diagnosed with another specified condition on the survey, so the parents have become less likely to select this category^1^. Notably, the questionnaire of NHIS underwent a significant redesign in 2019 to reduce the burden on respondents and align its content with other federal health surveys^30^. With these changes, some conditions will no longer be included in NHIS, such as cerebral palsy, seizures and stuttering or stammering, which could result in higher prevalence of other developmental delay in 2019 to 2021 (5.07–6.05%) than in 2015–2017(4.06%) and 2018 (4.02%)^1,30^. The prevalence of cerebral palsy, seizures and stuttering or stammering had not increased significantly in the decades before the 2019 questionnaire redesign^1,30^. But a true increase of other developmental delay in the study years assessed, which did not include cerebral palsy, seizures, and stuttering or stammering, cannot be ruled out. Supporting this notion, evidence revealed that the prevalence of other developmental delays in the NHIS survey increased from 3.57% in 2014 to 4.55% in 2016^13^. According to the NSCH report in 2016, less than a third of children aged 9 to 35 months had received standardized parent-completed developmental screening from healthcare professionals in the past year, and only one in five of those children received both screening and surveillance^31^. Extensive medical and developmental assessments are needed to detect developmental delays at an early age, and development outcomes for all children can be greatly improved through systematic screening and development surveillance for children.

### Co-occurring developmental disabilities

This survey showed that co-occurring LD & ID showed significant increase over time. In addition, this study also found that among the co-occurring developmental disabilities investigated in this study, the prevalence of co-occurring ADHD & LD, co-occurring LD & ASD, co-occurring ADHD & LD & ASD, co-occurring ADHD & LD & ID, and co-occurring LD & ASD & ID were high in children and adolescents. A whole population study from Scotland’s Census 2011 census reported that 21.7% of people with ID also had ASD and 18.0% of those with ASD also had an ID^32^. Approximately 4.7% of US children and adolescents aged 6 to 17 have ADHD without LD, 4.9% have LD without ADHD, and 3.7% have both conditions in NHIS 2004–2006^11^.

The increase in co-occurrence of LD and ID may be due to shared risk factors, cognitive similarities, challenges in early identification, and ambiguous diagnostic criteria^8,9,11,14^. Despite this overlap, it’s important to differentiate between LD and ID as their intervention needs vary^8,9,11,14^. Notably, one of the developmental disabilities may exacerbate or mitigate the typical symptoms of the other, which may complicate differential diagnosis or delay diagnosis and thus miss opportunity for treatment^9,14,32^. In addition, they may be associated in nature^33,34^, and future research should focus not only on exploring risk factors and etiology of co-occurring developmental disabilities, but also on perfecting diagnostic criteria for highly co-occurring conditions.

### Demographic and socioeconomic characteristics

There was significant difference across age, sex, race/ethnicity, family income levels, and geographic region for percentage among children and adolescents with any developmental disabilities in this study. Similar to previous surveys, the percentage of developmental disabilities did vary significantly by age, sex, race/ethnicity, or family income level^1,4,6,13,35^. Children and adolescents with a higher prevalence of any developmental disabilities were observed to be older (12–17 years old), boys, non-Hispanic, less educational level of family members, and lower family income to poverty ratio. These may be due to the fact that younger children are less likely to be screened or diagnosed^11,31^, biological or sex-specific manifestations^35,36^, and black individuals have improved access to health care in recent years^37^. In addition, children living in poverty experience adverse early experiences, such as inadequate stimulation or life stress, with exposure to multiple risks that affect brain development^38^. The brain develops rapidly in early life, and early childhood is a critical period for a child's cognitive, language, social, emotional and motor development^38^. Impaired brain development may lead to a higher susceptibility to developmental disabilities^38^. Children living in poverty have poorer grades in school or lower educational attainment, which ultimately lead to lower wages and income in adulthood^39^.

### Strengths and limitations

Our study has several notable strengths. Firstly, it benefits from a nationally representative sample derived from the US population, therefore enhancing the generalizability of our findings to a broader population. Secondly, our study has a large sample size and encompasses a diverse population with varying racial and ethnic backgrounds. This diversity allows us to examine disparities in the prevalence of developmental disabilities across different population characteristics.

However, there are also limitations. Initially diagnoses for all conditions were reported by parents or guardians, which may be subject to misreporting and recall bias, especially among parents of older children. Moreover, because of the cross-sectional design of the NHIS, direction of effects or inferences about causality cannot be made. Additionally, due to changes in the 2019 NHIS redesign, some conditions were removed, such as cerebral palsy, seizures and stuttering or stammering, and the overall prevalence of developmental disabilities may be impacted. Finally, the persistence of developmental disabilities varies depending on the conditions^1^. As parents reported lifelong diagnoses, some of the children included in the current analysis might no longer have diagnosable developmental disabilities.

## Conclusions

This study presents a comprehensive analysis of the prevalence and characteristics of children and adolescents who had ever been diagnosed with developmental disabilities, as reported by their parents, during the period from 2018 to 2021. We did not find an upward trend in the percentage of any developmental disability during this period, but given the high prevalence of developmental disabilities, it remains essential that we need to continue to monitor prevalence to understand potentially modifiable environmental risk factors and provide adequate resources for future diagnosis and treatment.

### Supplementary Information

Supplementary Information.

## Data Availability

The original contributions presented in the study are publicly available. The datasets analyzed during current study are available at NHIS online website: https://www.cdc.gov/nchs/nhis/index.htm.
