# Supplementary material for: Prevalence and trends of developmental disabilities among US children and adolescents aged 3 to 17 years, 2018–2021
Source: Sci Rep. 2023 Oct 12;13:17254. doi: 10.1038/s41598-023-44472-1 (PMC10570352; doi:10.1038/s41598-023-44472-1)

Prevalence and Trends of Developmental Disabilities among US Children and Adolescents aged 3 to 17 years, 2018-2021

Qian Li[1,+], Yanmei Li[1,+], Juan Zheng[2,+], Xiaofang Yan[1], Jitian Huang[1], Yingxia Xu[1], Xia Zeng[1], Tianran Shen[3], Xiaohui Xing[1], Qingsong Chen[4,*] & Wenhan Yang[1,*]

Supplementary Online Content

Table S. The Questions on ADHD, LD, ASD, ID, and Other Developmental Delay in NHIS 2018-2021

Figure S1. US Trends in the Prevalence of ADHD in Children and Adolescents Aged 3–17 Years, 2018-2021

Figure S2. US Trends in the Prevalence of LD in Children and Adolescents Aged 3–17 Years, 2018-2021

Figure S3. US Trends in the Prevalence of ASD in Children and Adolescents Aged 3–17 Years, 2018-2021

Figure S4. US Trends in the Prevalence of ID in Children and Adolescents Aged 3–17Years, 2018-2021

Figure S5. US Trends in the Prevalence of Other Developmental Delay in Children and Adolescents Aged 3–17 Years, 2018-2021

Table S. The Questions on ADHD, LD, ASD, ID, and Other Developmental Delay in NHIS 2018-2021

Variables	Survey Year	Survey Question
**ADHD**	2018	Has a doctor or health professional ever told you that [the sample child] had Attention Deficit Hyperactivity Disorder (ADHD) or Attention Deficit Disorder (ADD)?
	2019-2021	Has a doctor or other health professional ever told you that [the sample child] had Attention Deficit/Hyperactivity Disorder or ADHD or Attention-Deficit Disorder or ADD?
**LD**	2018-2021	Has a representative from a school or a health professional ever told you that [the sample child] had a learning disability?
**ASD**	2018	Has a doctor or health professional ever told you that [the sample child] had Autism, Asperger's disorder, pervasive developmental disorder, or autism spectrum disorder?
	2019-2021	Has a doctor or other health professional ever told you that [the sample child] had Autism, Asperger's disorder, pervasive developmental disorder, or autism spectrum disorder?
**ID**	2018	Has a doctor or health professional ever told you that [the sample child] had an intellectual disability, also known as mental retardation?
	2019-2021	Has a doctor or other health professional ever told you that [the sample child] had an intellectual disability, also known as mental retardation?
**Other Developmental Delay**	2018	Has a doctor or health professional ever told you that [the sample child] had any other developmental delay?
	2019-2021	Has a doctor or other health professional ever told you that [the sample child] had any other developmental delay?

Data source: NCHS, National Health Interview Survey, 2018-2021.

Abbreviation: ADHD, attention-deficit/hyperactivity disorder; LD, learning disability; ASD, autism spectrum disorder; ID, intellectual disability.

Figure S1. US Trends in the Prevalence of ADHD in Children and Adolescents Aged 3–17 Years, 2018-2021

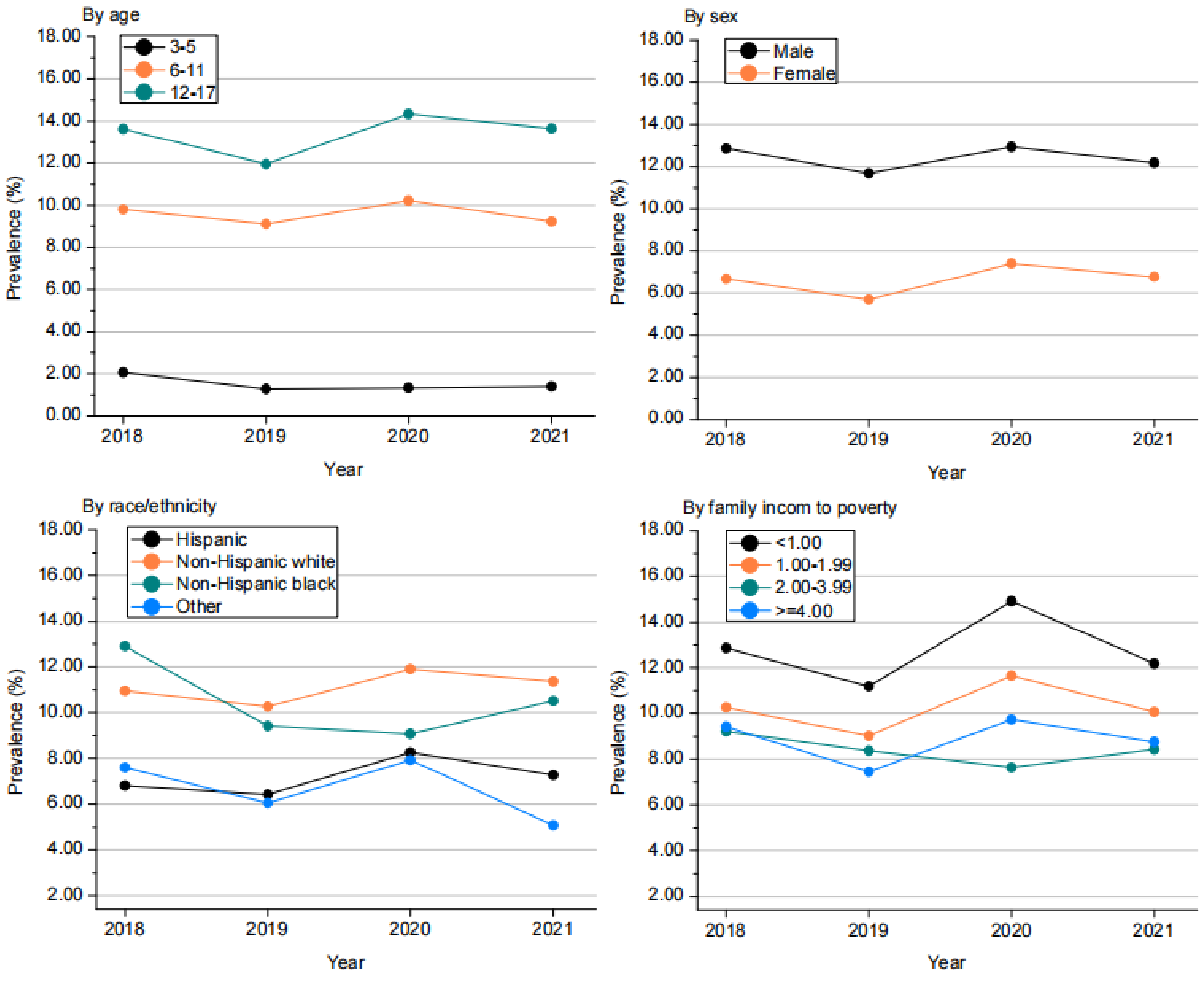

Figure S2. US Trends in the Prevalence of LD in Children and Adolescents Aged 3–17 Years, 2018-2021

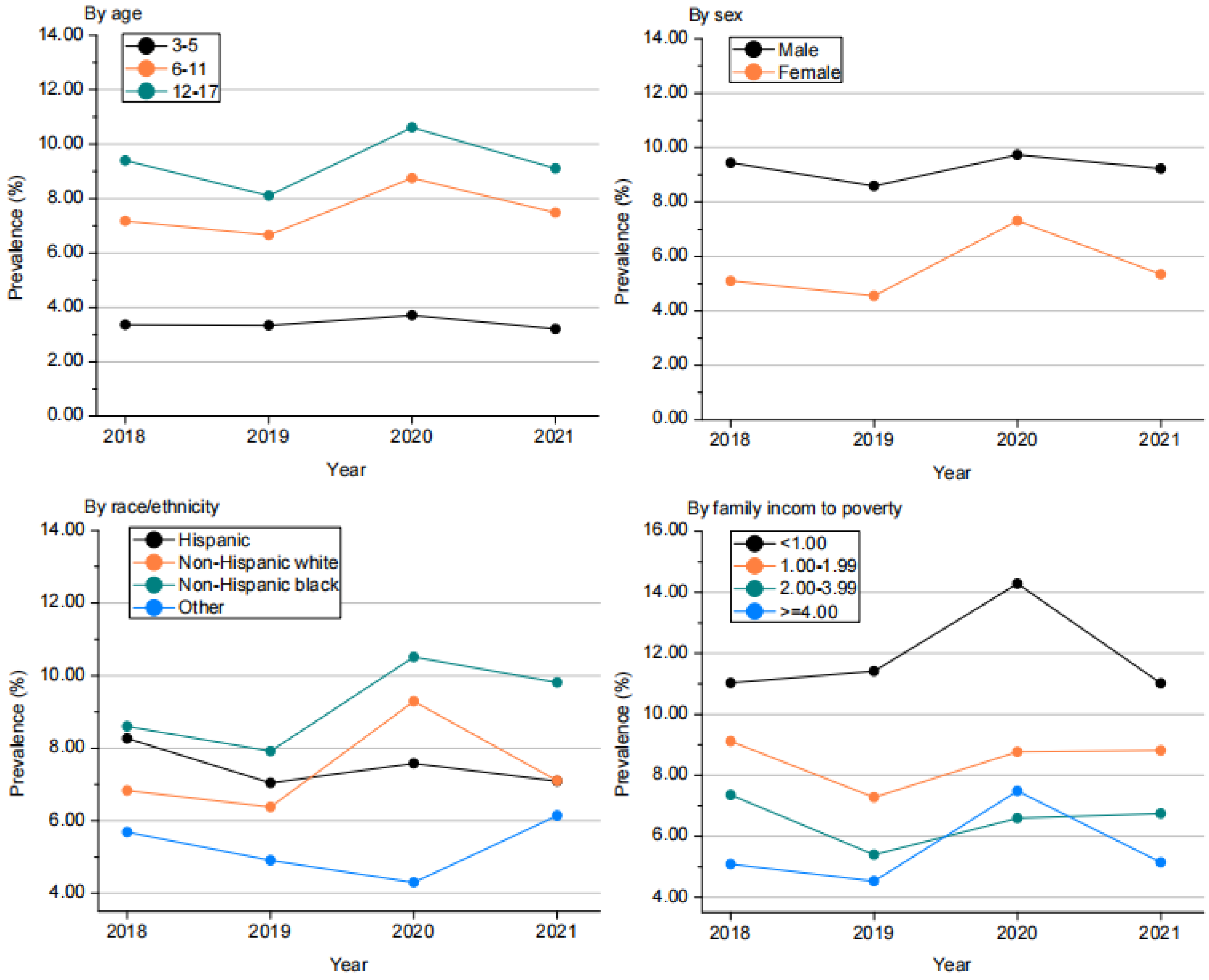

Figure S3. US Trends in the Prevalence of ASD in Children and Adolescents Aged 3–17 Years, 2018-2021

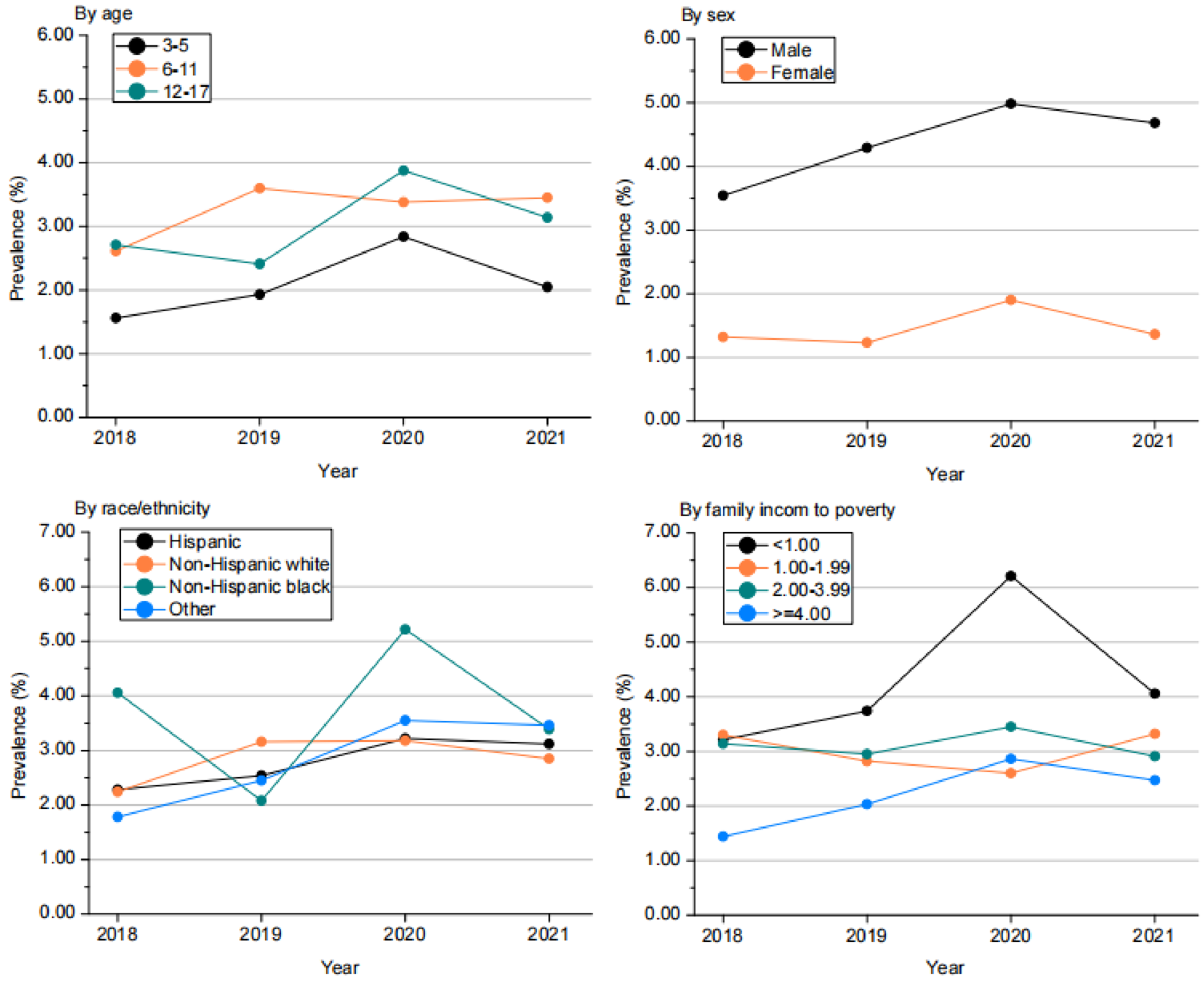

Figure S4. US Trends in the Prevalence of ID in Children and Adolescents Aged 3–17 Years, 2018-2021

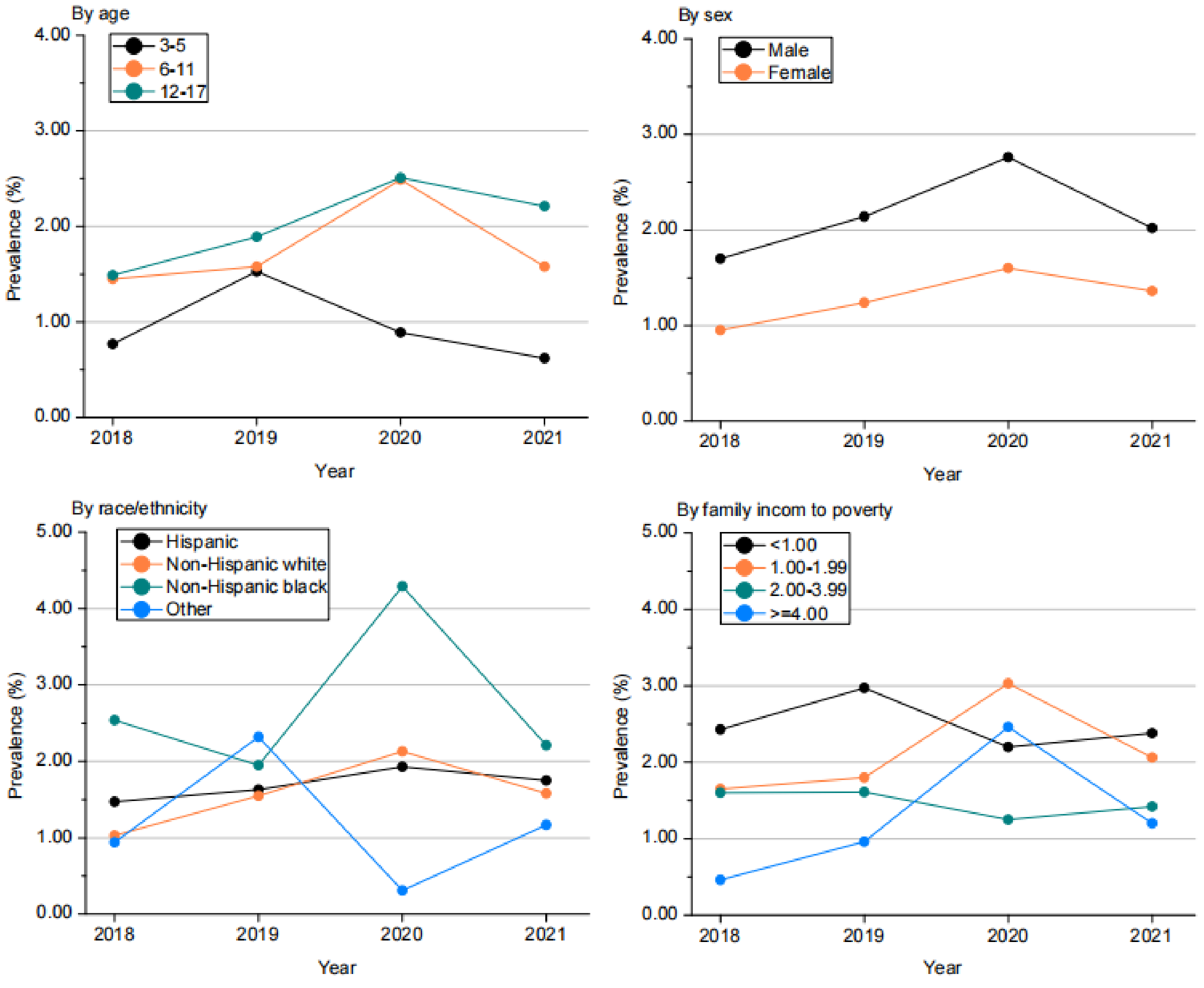

Figure S5. US Trends in the Prevalence of Other Developmental Delay in Children and Adolescents Aged 3–17 Years, 2018-2021

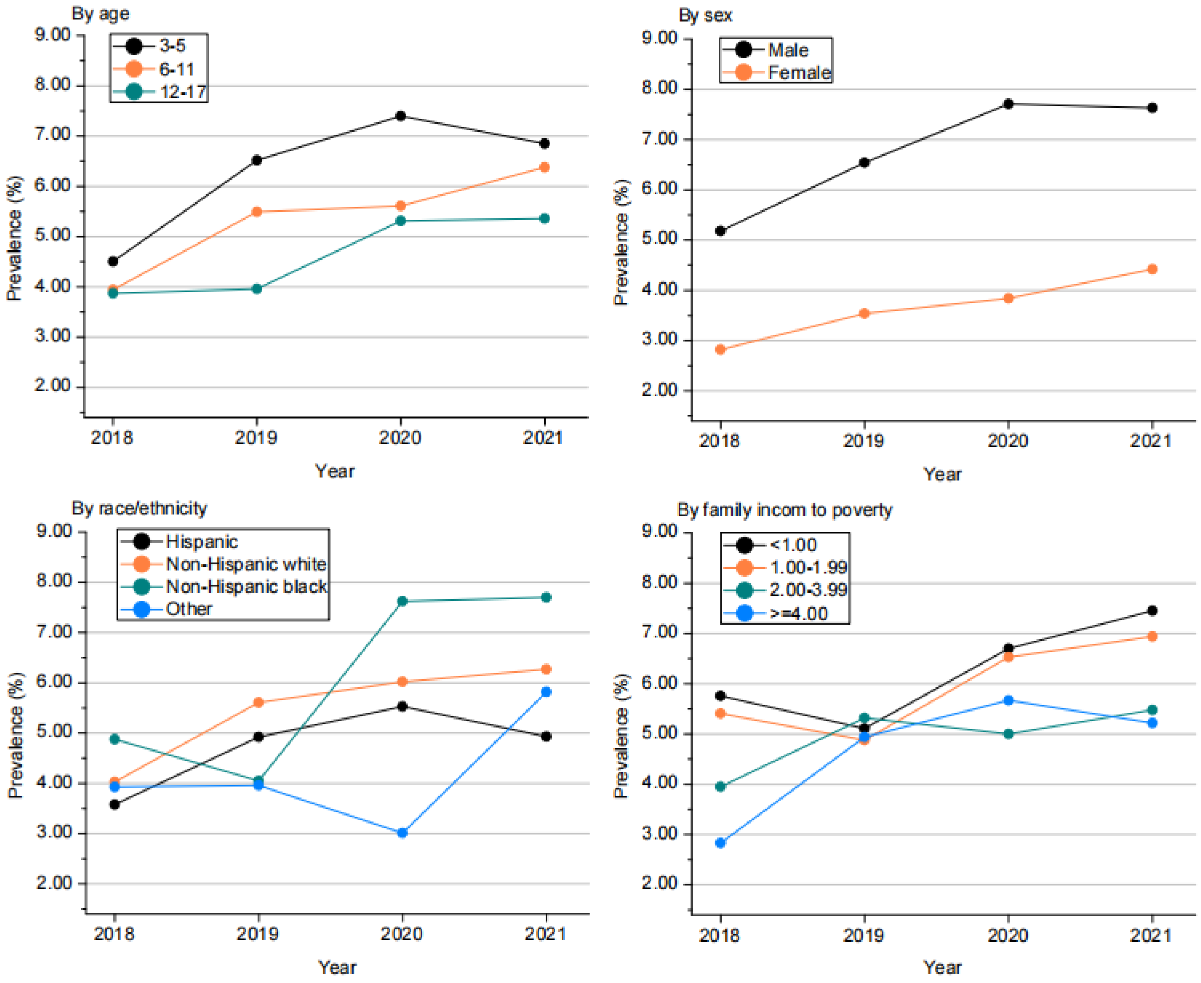

Supplement: Supplementary file 1 — Supplementary Information. [file 41598_2023_44472_MOESM1_ESM.pdf]
